# 18F-FDG simultaneous PET/MR findings of a malignant transformation and metastases of abdominal wall endometriosis

**DOI:** 10.1007/s00259-020-04761-7

**Published:** 2020-04-25

**Authors:** Haiyan Wang, Qiaoyi Xue, Yi Shou, Xing Chen, Zhiwen You, Jianmin Yuan, Jinli Gao, Jun Zhao

**Affiliations:** 1 Department of Nuclear Medicine, Shanghai East Hospital, Tongji University School of Medicine, Shanghai, China; 2 Central Research Institute, UIH Group, Shanghai, China; 3 Department of Pathology, Shanghai East Hospital, Tongji University School of Medicine, Shanghai, China

Abdominal wall endometriosis (AWE) arising from a surgical scar of cesarean delivery or other abdominopelvic operation, is a rare event with an incidence of 0.03 to 2% [1, 2]. Malignant transformation of AWE is an extremely rare disease, with few cases reported in the literature [3–5], and the atypical mechanism for progression to carcinoma remains unknown. The most frequent histological type of malignancy developing from extraovarian endometriosis is clear cell adenocarcinoma, followed by endometrioid adenocarcinoma [2, 6, 7]. Surgery and adjuvant chemotherapy are the primary treatment options. High mortality rate was found up to 50 months following diagnosis, with median survival time of 42 months after the diagnosis. Routine CT and MRI imaging are not sensitive in detecting malignancy and there is no specific clinical marker for malignant transformation. Whole-body 18F-fluorodeoxyglucose positron emission tomography/computed tomography (18F-FDG PET/CT) has been demonstrated to be a sensitive and well-established imaging modality for detection, staging/re-staging, and evaluation of therapy response of solid tumors. Simultaneous 18F-FDG positron emission tomography/magnetic resonance (PET/MR) has the potential to play an important role in identifying the malignancy and the evaluation of clinical staging because MRI has higher soft tissue contrast and no ionizing radiation exposure compared to CT.

We herein report a case of a 43-year-old woman with clear cell adenocarcinoma arising from malignant transformation of AWE 19 years after cesarean section. She was initially hospitalized for abdominal wall mass 3 months ago. The 10 × 5 cm of mass was palpated in the right lower abdomen. The mass was firm, tenacious, indolent to palpation, and had poor mobility. Abdominal needle biopsy was performed, and the histopathological results confirmed adenocarcinoma. Cancer antigen (CA) 125 level before the surgery was 26.20 IU/ml.

The patient was then sent for whole-body Fluorine-18 FDG positron emission tomography magnetic resonance (18F-FDG PET/MR) assessment for preoperative staging. A hybrid 3.0 T PET/MR scanner (uPMR 790, UIH, Shanghai, China) was used. Default clinical MRI sequences including T1w and T2w were scanned. 18F-FDG PET/MR imaging revealed a 9.4 × 4.6 × 4.8 cm solid, heterogenic mass in the abdominal wall on its exterior part, partially involving the right rectus abdominal muscle, with significantly increased FDG uptake (SUVmax = 9.61). Several enlarged retroperitoneal lymph nodes and left inguinal lymph nodes with high FDG uptake were also found in PET/MR-fused images (SUVmax = 4.25). There was no abnormal 18F-FDG uptake in the uterus and bilateral ovaries.

An extensive resection with bilateral appendectomy of uterus, pelvic lymph node cleaning, and reconstruction of the abdominal wall was performed. During the operation, a mass of 10 × 5 × 5 cm was found in the lower right rectus abdominis and between the peritoneum. The mass was firmly attached to the rectus abdominis, and it was difficult to separate it from healthy tissue. Multiple enlargement and fused lymph nodes were found in the bilateral iliac blood vessels and around the retroperitoneum. No obvious mass was found in the uterus and bilateral ovaries, and no effusion/infiltration was found in the abdominal and pelvic cavity.

Histopathological diagnosis was a stage IIIc clear cell adenocarcinoma originated in ectopic endometrial tissue, and 51 lymph nodes out of 69 ones was positive. Chemotherapy with taxol and carboplatin was then initiated.
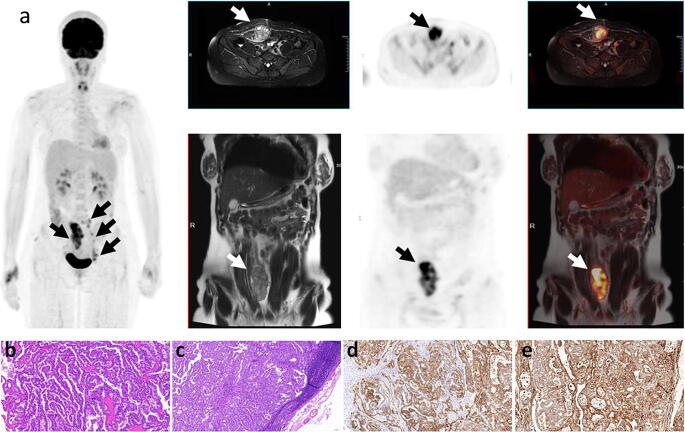


## References

[1] Bektas H, Bilsel Y, Sari YS (2010). Abdominal wall endometrioma: a 10-year experience and brief review of the literature. J Surg Res.

[2] Mihailovici A, Rottenstreich M, Kovel S (2017). Endometriosis-associated malignant transformation in abdominal surgical scar: a PRISMA-compliant systematic review. Medicine.

[3] Djakovic I, Vukovic A, Bolanca I (2017). Abdominal wall endometriosis eleven years after cesarean section: case report. Acta Clin Croat.

[4] Bats AS, Zafrani Y, Pautier P (2008). Malignant transformation of abdominal wall endometriosis to clear cell carcinoma: case report and review of the literature. Fertil Steril.

[5] Jiang M, Chen P, Sun L (2015). 18F-FDG PET/CT finding of a recurrent adenocarcinoma arising from malignant transformation of abdominal wall endometriosis. Clin Nucl Med.

[6] Sosa-Duran EF, Aboharp-Hasan Z, Mendoza-Morales RC (2016). Clear cell adenocarcinoma arising from abdominal wall endometriosis. Cirugia y Cirujanos.

[7] Marchand E, Hequet D, Thoury A, et al. Malignant transformation of superficial peritoneal endometriosis lesion. BMJ Case Rep. 2013. 10.1136/bcr-2012-007730. 10.1136/bcr-2012-007730 PMC3762544 23978494

